# Does Drug-Resistant Extrapulmonary Tuberculosis Hinder TB Elimination Plans? A Case from Delhi, India

**DOI:** 10.3390/tropicalmed5030109

**Published:** 2020-07-01

**Authors:** Sheelu Lohiya, Jaya Prasad Tripathy, Karuna Sagili, Vishal Khanna, Ravinder Kumar, Arun Ojha, Anuj Bhatnagar, Ashwani Khanna

**Affiliations:** 1 Lok Nayak Hospital, New Delhi 110002, India; dr.vishalkhanna@rediffmail.com (V.K.); dpsdllnc@rntcp.org (A.O.); stodl@rntcp.org (A.K.); 2 All India Institute of Medical Sciences, Nagpur 441108, India; ijay.doc@gmail.com; 3 International Union Against Tuberculosis and Lung Disease, The Union South East Asia Office, New Delhi 110016, India; KSagili@theunion.org; 4 All India Institute of Medical Sciences, New Delhi 110029, India; dpsdlndm@rntcp.org; 5 Rajan Babu Institute of Pulmonary Medicine and Tuberculosis, New Delhi 110009 India; dpsdlkcc@rntcp.org

**Keywords:** adverse drug reactions, unfavourable outcome, lymph node TB, bone TB, TB elimination, extrapulmonary tuberculosis

## Abstract

Extrapulmonary drug-resistant tuberculosis (DR-EPTB) poses a formidable diagnostic and therapeutic challenge.Besides associated with high morbidity, it is a major financial burden for the patient and the health system. In spite of this, it has often been neglected as it does not “pose” a visible public health threat. We study clinical profiles, treatment outcomes, and factors associated with unfavourable outcomes among DR-EPTB patients under programmatic settings in New Delhi, India, and evaluate how this could impact TB elimination. A retrospective analysis of all DR-EPTB patients registered at three nodal DR-TB centres in Delhi in 2016 was carried out. Of the 1261 DR-TB patients registered, 203 (16%) were DR-EPTB, with lymph nodes (118, 58%) being the most common site, followed by bone (69, 34%). Nearly 29% (*n* = 58) experienced adverse drug reactions with severe vomiting (26, 13 %), joint pain (21, 10%) and behavioral disorder (15, 7%). History of previous TB treatment was observed in a majority of the cases (87.7%). Nearly one-third of DR-EPTB cases (33%) had unfavourable treatment outcomes, with loss-to-follow-up (*n* = 40, 58%) or death (*n* = 14, 20%) being the most common unfavourable outcomes. In the adjusted analysis, weight band 31–50 kilograms (aRR = 1.8, 1.2–3.4) and h/o previous TB (aRR = 2.1, 1.1–4.8) were mainly associated with unfavourable outcomes. TB elimination efforts need to focus on all forms of TB, including DR-EPTB, leaving no one behind, in order to realise the dream of ending TB.

## 1. Introduction

Tuberculosis (TB) remains the top infectious killer, ranking above HIV/AIDS, with 10.0 million cases and 1.4 million deaths in 2018 [1]. *Mycobacterium tuberculosis* (MTB), the causative agent, usually affects the lungs (pulmonary TB/PTB). However, MTB may spread through lymphatic or hematogenous routes to virtually any organ in the body, resulting in extrapulmonary TB (EPTB). The most common sites of EPTB infection include peripheral lymph nodes, pleura, genitourinary sites, bones and joints, abdomen (peritoneum and gastrointestinal tract), and the central nervous system.

While EPTB has existed for millennia, pulmonary TB has remained the prime focus of global TB control programmes. EPTB is often less contagious than PTB, and is therefore overlooked even though it constitutes about 15% of all forms of TB, amounting to nearly 1 million incident cases notified in 2018, as per the WHO Global TB report [1]. Additionally, EPTB results in significant morbidity and mortality due to various diagnostic and therapeutic challenges that lead to delayed care. 

In the present era of HIV pandemic coupled with global emergence of multidrug-resistant TB (MDR TB) and extensively drug-resistant TB (XDRTB), drug-resistant EPTB (DR-EPTB) presents a real and new public health challenge that has yet to receive serious attention. While drug resistance in PTB has been extensively studied, DR-EPTB has been neglected. Several systematic reviews and individual patient meta-analysis have reported treatment outcomes of MDR-TB, without disaggregated outcomes of DR-EPTB [2,3,4,5]. The WHO MDR-TB update, as per the global TB report, shows treatment success of 55%, a death rate of 15%, 14% lost to follow up, 8% of failed treatment and 7% of the patients not evaluated [6]. However, there is little information on outcomes disaggregated by type of TB, especially DR-EPTB. Interestingly, the National Tuberculosis Program of India does not report treatment outcomes separately for PTB and EPTB in DRTB reports.

India continues to have the highest number of TB cases in the world, with nearly 2.69 million cases in 2018 [7]. It also features among the top 10 high MDR-TB burden countries, with nearly 130,000 MDR/RR-TB cases notified in 2018 [1]. The Revised National Tuberculosis Control Programme (RNTCP) has reported poor treatment outcomes of successive MDR-TB cohorts [5,8]. Previous studies in four large states of India also reported poor overall treatment outcomes (40%–56%) among DR-TB patients, with high rates of death and lost-to-follow-up (LTFU) [9,10]. However, there is scarce information in the country on the profile and treatment outcomes of DR-EPTB patients and their associated risk factors. While it may be expected that treatment outcomes and associated risk factors of DR-EPTB are different from those of DR-PTB, there is no scientific evidence to support this. 

India aims to eliminate TB by 2025; however, this goal will remain unachieved if EPTB, especially the drug-resistant cases, continues to be ignored [11]. Compared to the rest of the country, the situation is different in Delhi, with 42% EPTB among all TB cases, probably due to better availability of diagnostic services [8]. However, there are no estimates of the burden of DR-EPTB since disaggregate figures are not routinely reported in the programme. A previous study by Kant et al. in North India has reported a 13.4% prevalence of drug resistance among all EPTB cases [12]. Another study in Mumbai showed resistance in 29% of Mycobacterium isolates in extrapulmonary specimens [13]. A similar study from Chennai found that 37/189 (19%) of extrapulmonary TB specimens were multidrug-resistant, while one was extensively drug-resistant (XDR) [14].

A better understanding of this small yet significant group of patients is necessary to design effective interventions that might help reduce morbidity and mortality and improve treatment success rates. When the global community talks about TB elimination, it talks about TB per se; however, most interventions and strategies are focused on pulmonary TB. The strategy and milestones to end the global TB epidemic include all diagnosed TB cases and latent TB cases [7], hence we need specific interventions to focus on extrapulmonary TB, both sensitive and resistant. 

Indicators such as TB treatment coverage, treatment success rates, the percentage of TB-affected households that experience catastrophic costs due to TB and drug-susceptibility testing (DST) coverage for TB patients is difficult to measure, and sustainable development goals (SDGs) cannot be achieved without giving due importance to DR-EPTB [15].

To address these gaps, we carried out this operational research in order to study the demographics, clinical profiles, and treatment outcomes of patients with DR-EPTB registered at three selected DR-TB nodal centres in Delhi in 2016, and explored risk factors associated with unfavourable treatment outcomes. 

## 2. Methods

### 2.1. Study Design

This is a retrospective cohort study involving a record review of routine program data.

### 2.2. Setting

#### General Setting

Delhi is the capital of India inhabited by 18.6 million people, with a large number of migrants. It has one of the highest population densities of 11,320 persons per square km, and a literacy rate of 86% [16]. Delhi has the highest rate of TB notification in the country, probably due to better diagnostic facilities in tertiary care hospitals [17].

The Programmatic Management of Drug-Resistant TB (PMDT) services were launched in India in 2006 and obtained full geographical coverage in 2013. PMDT services started in Delhi in 2008 with a culture and drug susceptibility testing (C & DST) laboratory located at the state-owned Intermediate Reference Laboratory (IRL). Other tests, such as the cartridge-based nucleic acid amplification test (CBNAAT) and line probe assay (LPA), are also available at the IRL. PMDT services are provided through 25 chest clinics and four Nodal DR-TB centres in Delhi. Since 2018, all the chest clinics in Delhi have been designated as district DR-TB centres. 

**Figure 1:**
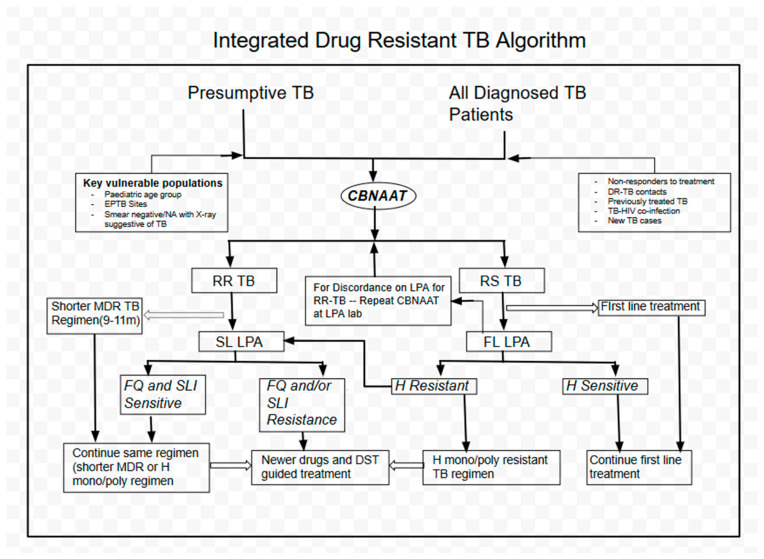
Diagnostic algorithm of drug-resistant tuberculosis as per PMDT India 2017. RR-TB, rifampicin-resistant tuberculosis; RS-TB, rifampicin-sensitive tuberculosis. SL-LPA, second line, line probe assay; FL-LPA, first line, line probe assay. FQ, fluoroquinolone; SLI, second line injectable; H, isoniazid; DRTB, drug-resistant TB; EPTB, extrapulmonary TB.

Microbiological confirmation of disease for DR-EPTB patients is preferred for diagnosis. This is done using either CBNAAT or culture or both. However, clinical diagnosis is also reached with the help of fine needle aspiration cytology (FNAC), histopathology findings, or interferon gamma release assays (IGRA), along with other signs and symptoms of TB, especially among those not responding to the WHO drug-sensitive ATT regimen. The patients are usually diagnosed at the district DR-TB centres or nodal DR-TB centres, and a sample, if available, is sent to the IRL along with a filled form requesting C&DST. Patients are started on the conventional MDR regimen at the Nodal DR-TB centres after pretreatment evaluation, as per NTP guidelines. Further follow-up and management is done at the district DR-TB centres. The diagnostic algorithm, which is common for both pulmonary and EPTB, is given in Figure 1 [18].

The conventional RNTCP regimen for MDR-TB is given to the patients with DR-EPTB, i.e., intensive phase with six drugs for 6–9 months (kanamycin, ofloxacin, ethionamide, cycloserine, pyrazinamide, ethambutol and pyridoxine), followed by a continuous phase with four drugs for 18 months (ofloxacin, ethionamide, cycloserine and ethambutol) as per RNTCP PMDT guidelines 2016 [7]. The patients found to be fluoroquinolone-resistant or resistant to other injectable drugs are started on the pre-XDRTB regimen (switched to high dose moxifloxacin and PAS).

Clinical monitoring is mainly based on clinical parameters such as weight gain, change in the size of lymph nodes/lesions, the appearance of new lymph nodes/lesions and monitoring of other EP sites located deep in the body by ultrasound, magnetic resonance imaging, computed tomography scan, and ESR (erythrocyte sedimentation rate). Surgery is considered in the absence of response to chemotherapy despite 6–9 months of treatment [18,19].

Treatment outcome definitions used by the RNTCP are given in Box 1. They are similar for pulmonary and extrapulmonary DR-TB patients. After the completed course of treatment, outcomes are assessed based on the response to treatment in terms of the resolution of symptoms and healing of lesions assessed through culture reports of specimens taken from discharging sinuses (if available) and investigation reports (e.g., ultrasonography, bone X-ray and magnetic resonance imaging). 

Regular monitoring of side effects of drugs is done by blood tests (complete blood count, serum urea, creatinine, electrolytes, blood glucose, alkaline phosphatases, transaminases, total bilirubin), audiometry, thyroid function tests, ocular examinations, ECG for QT prolongation and other tests, if needed.

Box 1 **Operational definitions for treatment outcomes in patients with multidrug-resistant TB (MDR TB).**

**Cure:** Treatment completed as recommended by the national policy without evidence of failure, and three or more consecutive cultures taken at least 30 days apart during CP are negative, including culture at the end of treatment.
**Treatment completed:** Treatment completed as recommended by the national policy without evidence of failure, but no record that three or more consecutive cultures taken at least 30 days apart are negative after the intensive phase.
**Treatment success:** This is a combination of cure plus treatment completed.
**Treatment failure:** Treatment terminated or a need for permanent regimen change of at least two or more anti-TB drugs in CP because of the lack of microbiological conversion by the end of the extended intensive phase or microbiological reversion in the continuation phase after conversion to negative or evidence of additional acquired resistance to FQ or SLI drugs or adverse drug reactions (ADR).
**Death:** A patient who dies for any reason during the course of treatment.
**Treatment lost-to-follow-up:** A patient whose treatment was interrupted for one month or more for any reasons prior to being declared as failed.
***Not evaluated:*** A patient for whom no treatment outcome is assigned.
***Regimen changed:*** A TB patient’s need for permanent regimen change of at least one or more anti-TB drugs prior to being declared as failed.
**Treatment stopped due to adverse drug reactions:** A patient who develops adverse drug reactions and cannot continue the M/XDR-TB treatment in spite of the management of adverse drug reactions as per the defined protocols and a decision has been taken by the DR-TB Centre committee to stop treatment.

### 2.3. Study Site

The study was conducted in three designated Nodal DR-TB centres under RNTCP in the state of Delhi.

### 2.4. Study Population

The study population included all DR-EPTB patients registered from 1 January 2016 to 31 December 2016 in the three selected nodal DR-TB centres in Delhi. These patients were admitted to a common TB hospital and initiated on a conventional MDR-TB regimen.

Those with associated pulmonary TB or on ITR (individualised treatment protocol) or XDR treatment regimen were excluded from the study; there were only three cases of associated pulmonary TB.

#### 2.4.1. Data Variables, Sources of Data and Data Collection

A list of all eligible DR-EPTB patients registered in 2016 at the selected nodal DR-TB centres was prepared. The principal investigator (SL) extracted data from the patient treatment cards and PMDT registers from September 2018 to February 2019 into a structured data collection instrument. 

Socio-demographic and clinical variables like PMDT TB number, date of registration, nodal DR-TB centre, age, sex, type of disease (primary/secondary MDR), site/s involved, history of previous TB treatment, site involved in previous TB episode, basis of diagnosis, resistance to drugs, comorbidities like HIV, diabetes, initial weight (in kilograms), final weight (in kilograms), adverse drug reaction, number of missed doses, treatment outcome (see Box 1) and date of outcome were included. Primary and secondary MDR was based on the previous TB treatment history.

#### 2.4.2. Data Analysis and Statistics

Data collected were double-entered and validated using EpiData version 3.1, and discrepancies were corrected by referring to the data collection forms or the original patient files. Data analysis was carried out using EpiData analysis version 2.2.2.183 (EpiData Association, Odense, Denmark) and STATA version 13.0. Number and proportion were used to summarise categorical variables, and mean (standard deviation) or median (interquartile ranges (IQR)), as applicable, were used to summarise continuous variables. A chi-square test was performed to find the association of various socio-demographic and clinical variables with the treatment outcome. Binomial regression was done to explore the predictors of unfavourable treatment outcomes after controlling for confounders. The strength of association was expressed using relative risks (RRs) and 95% confidence intervals (95% CI). Variables with *p* < 0.2 on univariable analysis were included in the final regression model. Unfavourable outcomes were defined as death, loss to follow-up, treatment failure, not evaluated, regimen change, or stopped treatment due to reasons other than adverse drug reactions. Favourable outcome was defined as treatment completed and cured.

## 3. Ethics Approval

Administrative approval was obtained from the State TB Office, Delhi, India. Ethics approval was obtained from the Ethics Advisory Group of the International Union Against Tuberculosis and Lung Disease, Paris, France. Names of patients were not captured. The PMDT registration number was used to identify patients. 

## 4. Results

### 4.1. Patient Characteristics

Of the total 1261 DR-TB patients registered in the three selected DR-TB sites in Delhi in 2016, 1058 (84%) were pulmonary and 203 (16%) were DR-EPTB cases, all of whom were included in the study. 

Most patients were female (111, 54.7%), aged 15–44 years (147, 72.4%). Around two-thirds of the patients (134, 66.0%) weighed less than 50 kilograms. Lymph node (118, 58.1%) was the most common site of involvement, followed by bone and joint (69, 34.0%). A large majority of patients had a previous history of TB (178, 87.7%). CBNAAT was the basis of diagnosis in 173 patients (85.2%) and LPA in 20 (9.9%) cases (Table 1**).**

### 4.2. Adverse Drug Reactions (ADRs)

Nearly 28.6% (*n* = 58) experienced at least one ADR, with severe vomiting (26, 12.8%), joint pain (21, 10.3%), behavioral disorder (15, 7.4%) and hearing loss (7, 3.4%) being the most commonly observed ADRs (Table 2).

### 4.3. Delay in Treatment Initiation

The median (IQR) number of days from diagnosis to registration for treatment was 15 [9,10,11,12,13,14,15,16,17,18,19,20,21,22,23,24,25] days, whereas from diagnosis to initiation of treatment was 14 days [8,9,10,11,12,13,14,15,16,17,18,19,20,21,22,23,24]. 

### 4.4. Treatment Outcomes

Overall treatment success was 66% (*n* = 134). Of the 69 (34%) patients with unfavourable treatment outcomes, most of them were due to LTFU (*n* = 40, 58.0%) or death (*n* = 14, 20.3%) (Table 3).

In the adjusted analysis, weight band 31–50 kilograms (aRR = 1.8, 1.2–3.4, *p*-value = 0.02), DR-TB centre (aRR = 1.5, 1.0–2.5, *p*-value = 0.05) and history of previous TB (aRR = 2.1, 1.1–4.8, *p*-value = 0.03) were significantly associated with unfavourable treatment outcomes (Table 4).

Stratified analysis was conducted to study the associations with unfavorable treatment outcomes in two different age groups (< 15 years and ≥ 15 years; Tables S1 and S2 in supplementary materials) Weight was not associated with unfavourable treatment outcomes among both children (< 15 years) and adults. Type of DR-TB centre was associated with the outcome among children and age ≥ 45 years was a significant predictor of unfavourable treatment outcome.

## 5. Discussion

The key findings of our study are: (i) one in every six registered DR-TB patients has DR-EPTB, (ii) lymph node is the most common site of involvement, followed by bone, in DR-EPTB patients, (iii) one-third of all DR-EPTB cases had unsuccessful treatment outcomes, and (iv) baseline weight, DR-TB centre and history of previous TB were significantly associated with unsuccessful treatment outcomes. 

Of all DR-TB patients registered, 16% were DR-EPTB cases. These figures are comparable to the prevalence of EP disease in drug-sensitive cases [20]. This is probably due to the availability and scale-up of rapid TB diagnostics (CBNAAT) in the region. With universal access to DST, DR-EPTB is an important and clinically challenging subgroup to tackle. A 10-year epidemiological study in China observed a higher proportion of MDR TB among patients with EPTB [21]. They observed a large increase in MDR TB, from 17.3% to 35.7%, for pleural TB cases. A similar high proportion of drug resistance has also been observed from extrapulmonary specimens in India [12,13,14]. The increasing drug resistance among EPTB highlights the need for drug susceptibility testing and the formulation of more effective regimens for extrapulmonary TB treatment.

Lymph node involvement is the most common EPTB, in general. We encountered a similar pattern in the DR-EPTB cases in the previous literature from the Netherlands (39%), the United States (40%), and the United Kingdom (37%) [11,22]. Pleural TB is the most prevalent form of extrapulmonary TB in Poland (36%) and Romania (58%), and bone TB (41%) is the most common site in China [21]. This study shows that bone TB (includes musculoskeletal TB) is the second most common site of DR-EPTB, accounting for nearly one-third of the cases. This finding is supported by other studies in the literature [23,24].

Nearly two-thirds of DR-EPTB cases (66%) had favourable treatment outcomes. This is better than the outcomes of the overall national DR-TB cohort (47%) notified between the 3rd quarter of 2014 to the 2nd quarter of 2015, which ranged between 36%–61% across all states [8]. Similarly, previous studies from India have also reported lower success rates among different DR-TB cohorts [5,9,10].

A recent study at a DR-TB centre in Mumbai reported a much higher completion rate of 82% among DR-EPTB patients, probably because it was a single centre study with different patient profiles and the use of a shorter regimen, which showed much better treatment completion rates [23].

A review found that the death rates among DR-EPTB patients were widely ranged from 0%–80% in different studies across the globe [11]. This wide variation in death rates could be due to various factors such as patient profiles, delayed diagnosis, comorbidities, severity of the disease, and type of regimen/treatment protocol. The present study reports 14 deaths (6.9%), probably because of treatment with suboptimal regimens. Culture DST or 2nd line LPA is not always possible, either due to insufficient clinical samples or inability to obtain samples from an inaccessible site, which renders resistance profiling difficult. Newer treatment options with newer drugs may be tried.

LTFU is common, which was seen among one-fifth of the patients. However, newer initiatives such as mobile adherence support, online web-based platform (Nikshay) for real-time reporting, efficient referral systems using Nikshay ID and newer treatment guidelines (all-oral H mono-resistant DRTB regimens, shorter MDR TB regimens, all-oral longer MDR TB regimens) could contribute to a decrease in LTFU.

The risk factors for unfavourable treatment outcomes help in stratifying patients for additional monitoring and improving outcomes. Patients with a previous history of TB have worse outcomes, which supports the findings in other studies [9,11]. This calls for more aggressive monitoring of treatment in such patients. 

Patients registered at DR-TB Centre 2 had a higher risk of unfavourable treatment outcomes, mostly LTFU or transferred out, because the centre receives a large number of patients from regions outside Delhi, who are eventually lost to follow-up. This calls for close monitoring, and better linkages and tracking of such patients.

A weight band between 31–50 kg at baseline was found to be significantly associated with unfavourable treatment outcomes compared to a weight band of < 30 kg. This has to be interpreted with caution as the role of unexplained confounders cannot be ruled out. A stratified analysis was conducted to study the association of weight bands on treatment outcomes in different age groups (children < 15 years and adults > 15 years). Although statistical significance was not seen, the proportion of outcomes among the 31–50 kg weight band was higher compared to other weight bands. Statistical nonsignificance could be due to small numbers and fewer numbers of outcomes in exposure categories, especially among children. This warrants a better understanding of the role of initial weight in determining treatment outcomes. We also need to explore whether a change in weight during the course of treatment or body mass index are better indicators compared to baseline weight in predicting outcomes. 

The strength of this study is that it was conducted within the routine programmatic setting in three designated Nodal DR-TB centres under RNTCP in Delhi. All the cases of DR-EPTB registered during the study period from three out of four nodal DR TB centres (covering 21 out of 25 chest clinics) in Delhi were included in the study without any exclusion, which covered nearly 90% of all such cases during the study period. This lends generalizability to the study findings. The study also adhered to Strengthening the Reporting of Observational studies in Epidemiology (STROBE) guidelines for conducting and reporting on observational studies [25]. There were some limitations as well. First, as this is a retrospective study using programmatic data, information on other possible predictors for TB treatment outcomes, such as socio-economic status, adherence to treatment, smoking and alcohol intake status, were not available. Second, a large majority of the cases were bacteriologically confirmed, thus indicating that many clinically diagnosed cases may have been missed. Third, the MDR-TB treatment regimens have changed in the last couple of years, such as the replacement of ofloxacin with levofloxacin or moxifloxacin, which might affect treatment outcomes. This requires similar analysis of the successive cohorts.

The study results have four important programmatic implications. First, high rates of death and LTFU among DR-EPTB patients need to be addressed urgently. Besides the risk factors identified in this study, some of which are non-modifiable, a shorter and easier-to-follow DR-TB treatment regimen with newer oral drugs such as bedaquiline or delamanid is probably the answer to reducing mortality and LTFU in this patient group. With the recent evidence from trials and large observational cohorts, the WHO stance has also departed from conventional treatment approaches for MDR TB in favour of shorter regimens with noninjectables [14,26,27]. However, newer drugs are being given only for pulmonary DRTB at present in India. The high LTFU could also well be due to the fact that a large chunk of the residents in the study area are mobile migrants. Delhi, being the capital city and a medical hub with modern health care facilities, receives patients from all over the country for diagnosis, who do not usually remain in the city to complete their treatment. Thus, close monitoring of the transfer-out-policy is necessary in order to understand the referral system, identify any loopholes within, and address them accordingly. Nikshay is a welcome step in this regard, wherein a unique ID is given to each patient to enable tracking. However, the objective of Nikshay ID is far from being achieved. More operational research is needed to identify the gaps in the referral mechanism and streamline the process to minimise leakages.

Second, low weight was also one of the risk factors for unfavourable treatment outcomes, which require tailored interventions to improve treatment outcomes for these patient sub-groups, especially those with poor weight at baseline and with a previous history of TB. The national program has initiated "Nikshay Poshan Yojana" which provides incentives for nutritional support to TB patients, which is a welcome step; however, studies have reported poor implementation of this [7]. Proper implementation of such schemes, with further customised options such as packaged groceries, would be more beneficial. 

Third, the proportion of DR EP-TB diagnosed clinically stands at a dismal 4% in this study. The diagnosed drug-sensitive EP-TB cases in Delhi constitute about 40%–45% of all TB cases, as per the India TB Report, while DR EP-TB is usually 15% to 20% of all DR-TB cases. It shows that we are probably missing clinically diagnosed EP-DRTB cases. Clinical nonresponders of EP-TB need to be reported too. In addition, there is over-reliance on bacteriological confirmation and drug sensitivity testing for the diagnosis of DR EP-TB, which is resulting in the underestimation of clinically diagnosed cases. Thus, diagnosis should be based on clinical judgement supported by culture/Xpert results, and not solely on bacteriological tests, as is common practice.

Fourth, those with a previous history of TB could be followed-up for at least two years to document relapse-free survival. For this, an aggressive strategy of follow-up and monitoring needs to be in place.

## 6. Conclusions

DR-EPTB constitutes a significant subgroup of the DR-TB cases that needs urgent attention. Every third DR-EPTB patient has unfavourable treatment outcomes, with high rates of LTFU that needs to be tackled if we are to realise the goal of ending TB. The use of shorter regimens and close monitoring/tracking of the migrant population and the transfer-out cases are required to minimise LTFU. Those with a previous history of TB need to be monitored closely for compliance to treatment. Further studies are needed to understand the operational reasons for the low proportion of clinically diagnosed DR EP-TB and explore the role of baseline weight or other proxy indicators, such as body mass index or weight gain during the treatment, in predicting treatment outcomes. 

## Figures and Tables

**Table 1:** Baseline demographic and clinical characteristics of drug-resistant extrapulmonary TB patients in Delhi in 2016 (*n* = 203).

	Characteristics	Number	(%)
**Sex**	Male	92	(45.3)
	Female	111	(54.7)
**Age (years)**	< 15	50	(24.6)
	15–44	147	(72.4)
	45 and above	06	(3.0)
**Weight (in kilograms)**	< 30	41	(20.2)
	31–50	93	(45.8)
	Above 50	69	(34.0)
**DR-TB Centre**	Centre 1	56	(27.6)
	Centre 2	59	(29.1)
	Centre 3	88	(43.3)
**Site of disease**	Peripheral lymph node	108	(53.2)
	Deep lymph node	10	(4.9)
	Brain	09	(4.4)
	Bone	69	(34.0)
	Pleural effusion	19	(9.4)
	Abdomen	10	(4.9)
	Heart	0	(0)
	Genital	02	(1.0)
**Basis of diagnosis**	CBNAAT	173	(85.2)
	Culture	2	(1.0)
	Line Probe Assay	20	(9.9)
	Clinical	8	(3.9)
**Drug Resistance**	HR	19	(9.4)
	Rifampicin	195	(96.1)
	Fluoroquinolone	05	(2.5)
	Other injectables	02	(1.0)
**History of TB**	Yes	178	87.7
	No	24	11.8
	Not recorded	01	(0.5)
**HIV status**	Negative	200	(98.5)
	Positive	03	(1.5)
**Diabetes**	Yes	05	(2.5)
	No	198	(97.5)
**Severe adverse reaction**	Yes	58	(28.6)
	No	145	(71.4)

TB: tuberculosis; HR: isoniazid, rifampicin CBNAAT: cartridge-based nucleic acid amplification test; DR-TB: drug-resistant tuberculosis. All CBNAAT-negative, culture-positive samples were subjected to second-line DST and put on LPA for first-line DST.

**Table 2:** Adverse drug reactions among drug-resistant extrapulmonary TB cases in Delhi in 2016 (*n* = 58).

Adverse Drug Reactions	Number	(%)
Severe vomiting	26	12.8
Behavior disorder	15	7.4
Hearing loss	7	3.4
Severe joint pain	21	10.3
Renal disturbance	03	1.5
Allergic reaction	04	2.0
Recurrent hepatitis	01	0.5
Gynaecomastia	02	1.0
Thyroid disturbance	02	1.0
Peripheral neuropathy	01	0.5
Vision loss	01	0.5
Others	02	1.0

Others include ocular disturbance, hemiparesis.

**Table 3:** Treatment outcomes of drug-resistant extrapulmonary TB cases in Delhi, 2016.

Adverse Drug Reactions	Number	(%)
Treatment success	134	66.0
Unfavourable treatment outcome	69	34.0
Death	14	6.9
Treatment failure	1	0.5
Lost to follow up	40	19.7
Regimen changed	3	1.5
Treatment stopped d/t ADR	0	0.0
Not evaluated	11	5.4

ADR = adverse drug reaction.

**Table 4:** Socio-demographic and clinical factors associated with unfavourable treatment outcomes among drug-resistant extrapulmonary TB cases in Delhi, 2016.

	Variables	Treatment Outcome			RR (95% CI)	*p*-Value	aRR (95% CI)	*p*-Value
		Total	Unfavourable					
		N	n	%				
**Sex**	Male	92	35	(38.0)	1.2 (0.8–1.8)	0.2	1.3 (0.9–1.9)	0.13
	Female	111	34	(30.6)	1.0		1.0	
**Age (years)**	≥ 15 years	153	57	(37.3)	1.6 (0.9–2.7)	0.08	1.6 (0.8–3.0)	0.14
	< 15 years	50	12	(24.0)	1.0		1.0	
**Weight (in Kilograms)**	< 30	41	8	(19.5)	1.0		1.0	
	31–50	93	37	(39.8)	2.0 (1.1–4.0)	0.02	1.8 (1.2–3.4)	**0.02**
	Above 50	69	24	(34.8)	1.8 (0.9–3.6)	0.09	1.6(0.8–3.0)	0.1
**DR-TB Centre**	Centre 1	59	16	(27.1)	1.0		1.0	
	Centre 2	88	37	(42.0)	1.6 (1.0–2.5)	0.06	1.5 (1.0–2.5)	**0.05**
	Centre 3	56	16	(28.6)	1.05 (0.6–1.9)	0.8	1.0 (0.7–2.0)	0.8
**Site of disease**	Others	104	38	(36.5)	1.2 (0.8–1.7)	0.4		
	Lymph node	99	31	(31.3)	1.0			
**Basis of diagnosis**	Others	30	10	(33.3)	1.0 (0.6–1.7)	0.9		
	CBNAAT	173	59	(34.1)	1.0			
**History of previous TB**	Yes	178	65	(36.5)	2.3 (1.0–5.7)	0.04	2.1 (1.1–4.8)	**0.03**
	No	25	4	16.0)	1.0		1.0	
**HIV status**	Negative	200	67	(33.5)	1.0	0.3		
	Positive	3	2	(66.7)	2.0 (0.9–4.5)			
**Diabetes**	Yes	5	3	(60.0)	1.8 (0.9–3.8)	0.2	1.9 (0.9–3.4)	0.18
	No	198	66	(33.3)	1.0		1.0	
**Severe adverse reaction**	Yes	58	16	27.6	1.0	0.2	1.0	
	No	145	53	36.6	1.3 (0.8–2.1)		1.4 (0.9–2.2)	0.15

TB: tuberculosis; HR: isoniazid, rifampicin; ADR: adverse drug reaction; CBNAAT: cartridge-based nucleic acid amplification test.

## Supplementary Materials

The following are available online at https://www.mdpi.com/2414-6366/5/3/109/s1.
