# Supplementary material for: Does Drug-Resistant Extrapulmonary Tuberculosis Hinder TB Elimination Plans? A Case from Delhi, India
Source: Trop Med Infect Dis. 2020 Jul 1;5(3):109. doi: 10.3390/tropicalmed5030109 (PMC7558170; doi:10.3390/tropicalmed5030109)
Supplement: Supplementary file 1 [file tropicalmed-05-00109-s001.pdf]

Tropical Medicine and Infectious Disease

MDPI

Supplementary

Does Drug-Resistant Extrapulmonary Tuberculosis Hinder TB Elimination Plans? A Case from Delhi, India

Sheelu Lohiya [1,*], Jaya Prasad Tripathy [2], Karuna Sagili [3], Vishal Khanna [1], Ravinder [4], Arun Ojha [1], Anuj Bhatnagar [5] and Ashwani Khanna [1]

1. Lok Nayak Hospital, New Delhi 110002, India; dr.vishalkhanna@rediffmail.com (V.K.); dpsdllnc@rntcp.org (A.O.); stodl@rntcp.org (A.K.)
2. All India Institute of Medical Sciences, Nagpur 441108, India; ijay.doc@gmail.com
3. International Union Against Tuberculosis and Lung Disease, The Union South East Asia Office, New Delhi 110016, India; KSagili@theunion.org
4. All India Institute of Medical Sciences, New Delhi 110029, India; dpsdlndm@rntcp.org
5. Rajan Babu Institute of Pulmonary Medicine and Tuberculosis, New Delhi 110009 India; dpsdlkcc@rntcp.org

* Correspondence: dtodllnc@rntcp.org

Received: 11 March 2020; Accepted: 28 May 2020; Published: date

Table S1. Socio-demographic and clinical factors associated with unfavorable treatment outcome among drug resistant extra-pulmonary TB cases in Delhi aged < 15 years, 2016.

Variables		Treatment Outcome: Total N	Unfavorable n	%	RR (95% CI)	p value
Sex	Male	21	5	(23.8)	1.0 (0.4–2.7)	0.9
	Female	29	7	(24.1)	1.0	
Weight (in Kilograms)	< 30	36	7	(19.4)	1.0	
	31–50	14	5	(35.7)	1.8 (0.7–4.8)	0.3
DR-TB Centre	Centre 1	15	1	(6.7)	1.0	
	Centre 2	21	5	(23.8)	3.6 (0.5–27.5)	0.3
	Centre3	14	6	(42.9)	6.4 (1.4–26.9)	0.02
Site of disease	Others	22	3	(13.6)	0.4 (0.2–1.4)	0.13
	Lymph node	28	9	(32.1)	1.0	
Basis of diagnosis	Others	5	0	(0.0)	--	0.32
	CBNAAT	45	12	(26.7)	1.0	
History of previous TB	Yes	40	11	(27.5)	2.7 (0.4–18.9)	0.24
	No	10	1	(10.0)	1.0	
HIV status	Negative	48	11	(22.9)	1.0	
	Positive	2	1	(50.0)	2.2 (0.5–9.5)	0.4
Severe adverse reaction	Yes	7	1	(14.3)	0.6 (0.2–3.7)	0.5
	No	43	11	(25.6)	1.0	

RR: Relative Risk; DR-TB: Drug Resistant Tuberculosis; CBNAAT: Cartridge Based Nucleic Amplification Test; HIV: Human Immunodeficiency Virus.

Table S2. Socio-demographic and clinical factors associated with unfavorable treatment outcome among drug resistant extra-pulmonary TB cases in Delhi aged ≥15 years, 2016.

Variables		Treatment Outcome: Total N	Unfavorable n	%	RR (95% CI)	p value
Sex	Male	71	30	(42.3)	1.3 (0.9–1.9)	0.2
	Female	82	27	(32.9)	1.0	
Age (years)	15–44 years	147	52	(35.4)	1.0	

	> 45 years	6	5	(83.3)	2.4 (1.6–3.6)	0.02
**Weight (in Kilograms)**	< 30	5	1	(20.0)	1.0	
	31–50	79	32	(40.5)	2.0 (0.3–12.0)	0.6
	Above 50	69	24	(34.8)	1.7 (0.3–10.0)	0.6
**DR-TB Centre**	Centre 1	44	15	(34.1)	1.0	
	Centre 2	67	32	(47.8)	1.4 (0.9–2.3)	0.1
	Centre 3	42	10	(23.8)	1.4 (0.7–2.8)	0.8
**Site of disease**	Others	82	35	(42.7)	1.4 (0.9–2.1)	0.1
	Lymph node	71	22	(31.0)	1.0	
**Basis of diagnosis**	Others	25	10	(40.0)	1.1 (0.6–1.8)	0.7
	CBNAAT	128	47	(36.7)	1.0	
**History of previous TB**	Yes	138	53	(38.4)	1.4 (0.6–3.4)	0.4
	No	15	4	(26.7)	1.0	
**HIV status**	Negative	152	56	(36.8)	1.0	0.3
	Positive	1	1	(100)	2.7 (2.2–3.3)	
**Diabetes**	Yes	5	3	(60.0)	1.6 (0.8–3.5)	0.3
	No	148	54	(36.5)	1.0	
**Severe adverse reaction**	Yes	51	15	(29.4)	0.7 (0.4–1.2)	0.2
	No	102	42	(41.2)		

RR: Relative Risk; DR-TB: Drug Resistant Tuberculosis; CBNAAT: Cartridge Based Nucleic Amplification Test; HIV: Human Immunodeficiency Virus.

© 2020 by the authors. Submitted for possible open access publication under the terms and conditions of the Creative Commons Attribution (CC BY) license (http://creativecommons.org/licenses/by/4.0/).
